# A deep learning framework combined with word embedding to identify DNA replication origins

**DOI:** 10.1038/s41598-020-80670-x

**Published:** 2021-01-12

**Authors:** Feng Wu, Runtao Yang, Chengjin Zhang, Lina Zhang

**Affiliations:** School of Mechanical, Electrical and Information Engineering, Shandong University at Weihai, Weihai, 264200 China

**Keywords:** Bioinformatics, Machine learning

## Abstract

The DNA replication influences the inheritance of genetic information in the DNA life cycle. As the distribution of replication origins (ORIs) is the major determinant to precisely regulate the replication process, the correct identification of ORIs is significant in giving an insightful understanding of DNA replication mechanisms and the regulatory mechanisms of genetic expressions. For eukaryotes in particular, multiple ORIs exist in each of their gene sequences to complete the replication in a reasonable period of time. To simplify the identification process of eukaryote’s ORIs, most of existing methods are developed by traditional machine learning algorithms, and target to the gene sequences with a fixed length. Consequently, the identification results are not satisfying, i.e. there is still great room for improvement. To break through the limitations in previous studies, this paper develops sequence segmentation methods, and employs the word embedding technique, ‘Word2vec’, to convert gene sequences into word vectors, thereby grasping the inner correlations of gene sequences with different lengths. Then, a deep learning framework to perform the ORI identification task is constructed by a convolutional neural network with an embedding layer. On the basis of the analysis of similarity reduction dimensionality diagram, Word2vec can effectively transform the inner relationship among words into numerical feature. For four species in this study, the best models are obtained with the overall accuracy of 0.975, 0.765, 0.885, 0.967, the Matthew’s correlation coefficient of 0.940, 0.530, 0.771, 0.934, and the *AUC* of 0.975, 0.800, 0.888, 0.981, which indicate that the proposed predictor has a stable ability and provide a high confidence coefficient to classify both of ORIs and non-ORIs. Compared with state-of-the-art methods, the proposed predictor can achieve ORI identification with significant improvement. It is therefore reasonable to anticipate that the proposed method will make a useful high throughput tool for genome analysis.

## Introduction

Since the theory of DNA replication was proposed, the bioscience has been undergoing profound changes, which greatly motivates various studies based on the DNA replication, including cell growth and cell division. As a rigorous biological process starting at ‘ORI’ (origin of replication), DNA replication can generate two identical daughter strands by unwinding the parental template strands with the semiconservative replication strategy^1–4^. To keep normal cell functions and inherit a complete set of genomic information, DNA replication is activated only once per cell cycle^5^. It is worth noticing that the DNA replication is closely related to the transmission of genetic information^6^. For instance, previous study indicated that DNA replication frequently occurs in the downstream of gene sequences and less likely around transcription initiation sites^7^. Therefore, it is essential to understand the role and mechanism of DNA replication. In this regard, the accurate identification of ORIs will provide insights into the potential biological roles of DNA replication in the spatial and temporal regulation of the gene transcription and gene expression.

A series of biological technologies have been developed to precisely identify ORIs. Chromatin Immunoprecipitation (ChIP)^8^ is to fix the protein-DNA complex in a living cell state, and randomly cut it into small chromatin fragments, then precipitate the complex by immunological methods to enrich the DNA fragments bound by the target protein. The information of protein-DNA interaction is obtained by purification and detection of the target fragment. With the development of modern sequencing technology, ORIs can also be obtained by sequencing the immunoprecipitated complex, called ChIP-seq^9^. Above all, CHIP is adopted to specifically enrich the DNA fragments bound by the target protein. Then, the enriched DNA fragments are subjected to high-throughput sequencing. Finally, millions of sequence tags are accurately located on the genome to obtain DNA segment information among which interacts with histones, transcription factors.

Drawing on the aforementioned sequencing technology, many ORI related theories and principles have been developed, which greatly facilitate the genome research of various species. In different species, ORIs have both common structural features and certain differences. For instance, some original features of ORIs in Arabidopsis thaliana are shared with animal cells, and it also has characteristics typically existed in plant cells^10^. The ORIs in mouse and drosophila genome contain abundant ‘AT’ in $\alpha$-strand or ‘TA’ in its complementary strand^11^. Besides, the ORIs in *S. cerevisiae* is formed by 3 domains named domain A, domain B and domain C^12^. Each of the three domains has its special motif and function as elaborated in^13,14^. In the *S. cerevisiae* genome, there are over 12,000 conserved sequences where only 400 contain ORIs^15^. In fact, the replication mechanism of *S. cerevisiae* is investigated most extensively and deeply, and the first recognized eukaryotic ORI sequence is from *S. cerevisiae*^16^. Generally, there are some striking differences in prokaryotes and eukaryotes in terms of DNA replication. Prokaryotes have a single circular DNA molecule^17^, with only one ORI due to their simple cell structure and the relatively small number of genomic bases compared with eukaryotes. To complete the complex DNA replication of the entire genome in a fast and efficient way, the large number of DNA bases and the restriction of dinucleotides incorporation in eukaryotes allow simultaneous replication from multiple ORIs^18^. For instance, about 30,000 ORIs are activated during cell division in mice.

In the post-genome era, the emergence of a large number of genomic sequences has highlighted the defects of experimental methods on time-consuming and cost. Against this background, computational methods to efficiently predict the ORIs are urgently needed. For bacteria, there are many sophisticated methods that can accurately identify their ORIs. ‘ORI-finder’ is an online system based on the analysis of nucleotide composition asymmetry using Z-curve method, the distribution of DnaA boxes and the occurrence of genes frequently close to ORIs^19^. On the basis of the ‘ORI-finder’, the analysis of the distribution of the origin recognition boxes (ORB) elements identified by the Find Individual Motif Occurrences (FIMO) software is incorporated to compose a more effective online predictor called ‘ORI-finder 2’^20^. In addition, Shah et al. suggested a correlation based approach which directly considers the spatial positioning of a specific base in genomic region to achieve excellent identification results in lower organisms^21^. However, for eukaryotes, with multiple ORIs, it is difficult to achieve a satisfactory and accurate identification of ORIs.

In the past few years, several desired identification results have been obtained to identify ORIs of eukaryotes. Wang et al.^22^ adopted Z-curve theory to convert the DNA sequence to a geometrical curve, and proposed a windowless approach to calculate and segment the AT-rich region along the DNA sequence, which achieved a stable identification result of ORIs. Chen et al.^23^ conducted the analysis of two structural characteristics, namely, DNA bendability and cleavage intensity around ORIs in the *Saccharomyces cerevisiae* genome, then developed a support vector machine (SVM) based model for ORI prediction, which achieved a satisfying accuracy of 85.86% under the jackknife cross validation. With the development of bioinformatics and NLP, increasing attention has been paid to use sequence information to realize the identification of ORIs. By extending the pseudo amino acid composition (PseAAC)^24^ from protein into the realm of DNA, Li et al. employed the pseudo k-tuple nucleotide composition (PseKNC) to encode gene sequences which can reflect the intrinsic correlation between local/global features and the ORIs. The SVM was utilized to operate the prediction and the accuracy of ORIs predicted in the *S. cerevisiae* genome with the proposed model called ‘iORI-PseKNC’ reached to 83.72%^25^. Dao et al. aimed to enhance the prediction capability in recognizing yeast ORIs. 90 physicochemical properties were incorporated into PseKNC to characterize the DNA sequences. Meanwhile, F-score^26^ and mRMR^27^ were utilized to optimize features. Then a SVM based model called ‘iORI-PseKNC2.0’ was developed to perform classification with an accuracy of 87.79%^28^. Xiao et al. successfully incorporated dinucleotide location-specific propensity into PseKNC and used Random Forest^29^ classifier to form a predictor called ‘iROS-gPseKNC’, which has a pretty high accuracy of 98.03% and other indexes are also close to 100%^30^. Zhang et al. demonstrated that the integration of dinucleotide physicochemical properties with the pseudo nucleotide composition is an effective way to improve the prediction performance of human ORIs, and the Random Forest classifier was used to form the predictor, called ‘iOri-Human’^31^. The latest method proposed by Do et al.^32^ is a hybrid identification system incorporating fusion features extracted by FastText^33^ and PseKNC with XGBoost^34^, which achieved an accuracy of 89.51% in *Saccharomyces cerevisiae*.

The commonality among the aforementioned predictors is that they aimed to identify ORIs of one species and could only be used to identify tiny parts (250 or 300 dp) of the replication origins. To overcome the limitation, some methods were proposed to identify ORIs of yeast species. A recent method, ‘iRO-3wPseKNC’, incorporated the ‘GC asymmetry bias’ into the ORIs prediction as the feature by three-window-based PseKNC. It is worthy to mention that iRO-3wPseKNC based on Random Forest performs well in four yeast species, and even gets the accuracy of 96.5% in *Schizosaccharomyces pombe*^35^. In addition, it is the first predictor which can identify ORIs sequences with unfixed length. On the basis of the ‘iRO-3wPseKNC’, to reflect the uneven distribution of G and C, Liu et al. proposed a predictor called ‘iRO-PseGCC’ by capturing the GC asymmetry bias and incorporating the GC Skew into the concept of PseKNC^36^.

All the aforementioned predictors have their own advantages and significantly enhance the development of ORIs identification, but they still have some limitations. Firstly, previous methods only took the local DNA sequence information into account and ignored the global DNA sequence information to capture long-range interactions that are close in the three-dimensional space, but far from each other in their sequence positions. Secondly, the performance of previous methods relys heavily on the hand-crafted features, which may be limited by the lack of experience and domain knowledge. To take full advantage of the rapid expansion of gene sequences, a more robust, automatic framework to extract sequence-dependent features is desired. Thirdly, previous methods are developed by traditional machine learning^37^ methods, i.e., shallow models for supervised learning, such as SVM and Random Forest. In addition, feature extraction and classification are considered as two processes, which may limit the prediction performance.

Deep learning has received extensive attention in the boom of artificial intelligence as a cutting-edge machine learning method. It generally refers to a collection of deep neural network algorithms that has multiple hidden layers which can learn hierarchical representations and detect complex patterns by digging deep features from datasets. Amongst a set of deep neural networks, convolutional neural network (CNN) is extensively employed in both academia and industry. Traditional CNNs are not suitable to address sequences identification tasks. Therefore, CNNs with some special architectures are gradually popular to be employed to perform classification tasks in bioinformatics^38,39^. Inspired by the excellent performance of deep learning in image processing^40,41^, natural language processing^42,43^ and many other fields^44,45^, a deep learning framework is constructed in this study by a CNN with an embedding layer to automatically learn a suitable representation of the raw data, discover high-level features, and improve ORI identification performance over traditional models.

It is worth noticing that bioinformatics and natural language processing (NLP) greatly promote and benefit from each other. Similar to the complex grammatical and semantic structure in natural languages, nucleotide composition and sequence structure determine the motif and function of gene sequences^46^. At present, it is popular to consider nucleotides as ‘words’ which are sometimes ambiguous in biological sequences (DNA, RNA, proteins), unlike the regular concept of ‘words’ in natural languages. For instance, Le et al. spliced the word vector of 10-gram as the representation of a DNA sequence and input into 1D-CNN to detect promoters^47^. Do et al. adopted Fasttext to train the word vectors of 3-gram, and constructed a DNN framework with the input of the pre-trained word vectors^48^. Word embedding technology is a general term for language models and representation learning technologies in the field of natural language processing. Conceptually, it refers to allowing machines to learn distributed representations of words by embedding a high-dimensional space with the number of all words in a low-dimensional continuous vector space. Before the advent of word embedding technology, one-hot representation is a traditional method, but one-hot is too sparse to reflect the interrelationship between words. In addition, the principal component analysis (PCA)^49^ and the T-distributed neighborhood embedding algorithm (T-SNE)^50^ can be adopted to further reduce the dimension of the distributed representation in the word embedding space, thereby realizing the visualization of word embedding and word meaning induction. In view of this, word embedding technology is utilized in this paper to realize the distribution representation.

**Figure 1:**
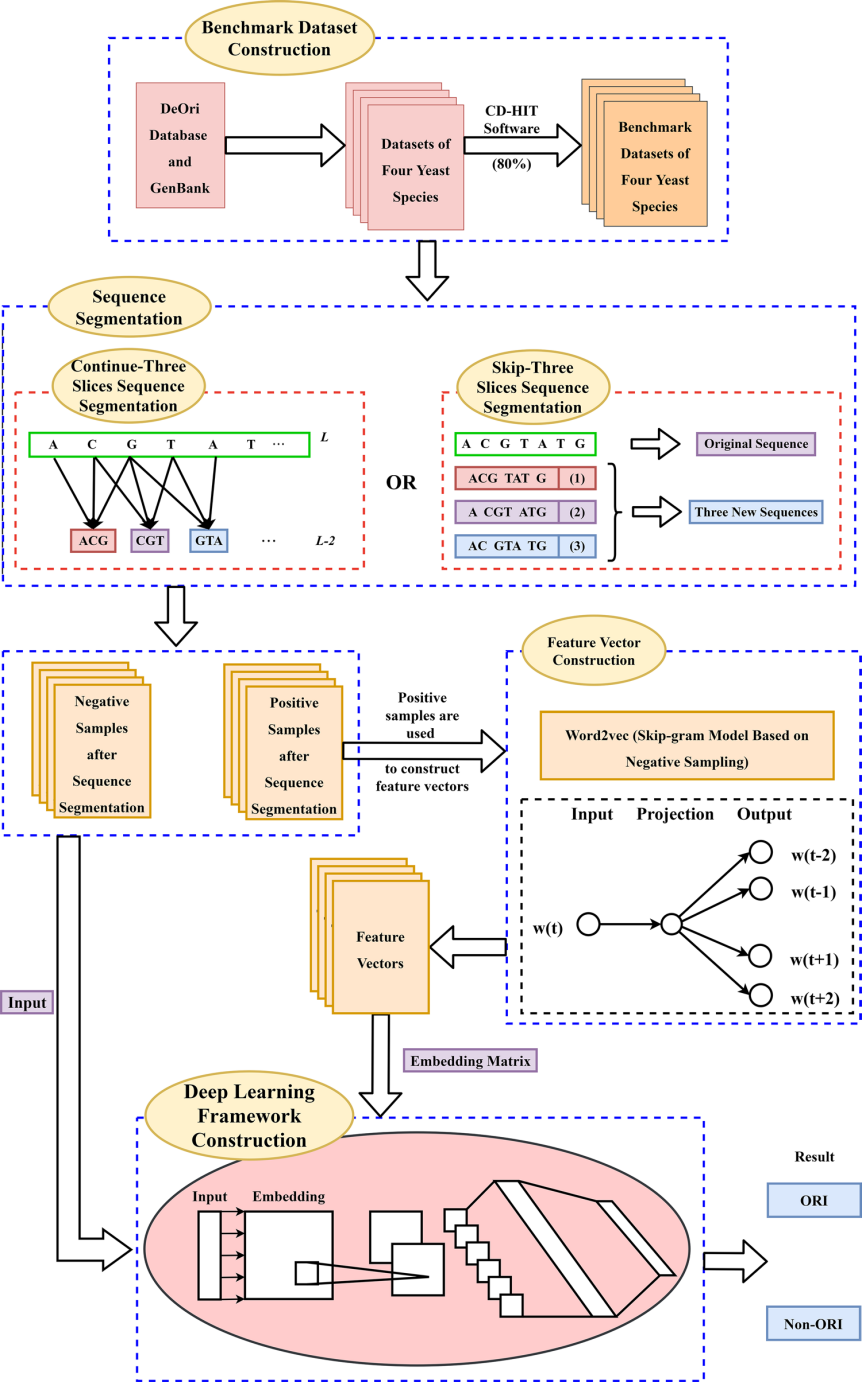
The overall workflow of the proposed method.

As shown in Fig. 1, the proposed method is organized into the following four main steps, i.e. benchmark dataset construction, sequence segmentation, feature vector construction and deep learning framework construction. Specifically, the genomic locations of replication origins and corresponding gene sequences of four yeast species are derived from the database. To avoid data imbalance and reduce homologous bias, the benchmark datasets of four species are obtained after removing high homologous samples and short samples. Subsequently, by a sliding window with 3 bases and a step of 1, each DNA sequence in the benchmark dataset is scanned across from the left to the right to generate a series of trinucleotides. Similarly, by a sliding window with 3 bases and a step of 3, one sequence could be divided into three new sequences with the same label that are adopted to rebuild the feature vectors. Then, ‘Word2vec’ in the field of NLP is employed to map trinucleotides from an original space to a new multidimensional space. Finally, a deep learning framework is constructed by a CNN with an embedding layer to identify ORIs, and the proposed method is compared with the existing methods on the same dataset by the 10-fold cross validation.

## Results and disccusion

### Analysis of feature spaces

To analyze the general sequence-based characteristics of ORIs and non-ORIs, visualizing feature vectors are needed to investigate whether feature vectors constructed by the Word2vec can depict the inner properties of DNA sequences. It is worth noting that the feature vectors of the same word trained by Word2vec are quite not invariant for different iterations, due to the differences on the vector initialization and the back propagation of network parameters.

**Figure 2:**
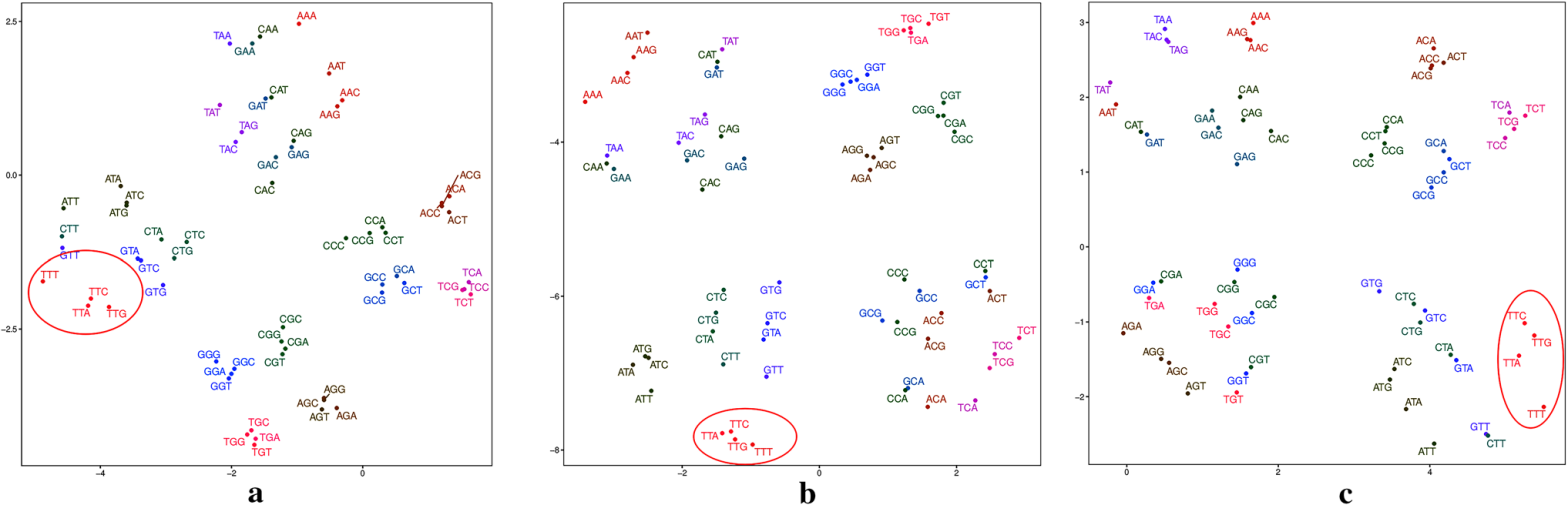
The 2-dimensional feature space generated by the *t-SNE* algorithm in dataset $S_{1}$ using Continuous-TSSS. (**a**) The first training result. (**b**) The second training result. (**c**) The third training result.

As shown in Fig. 2, a 2-dimensional feature space in the dataset $S_{1}$ can be obtained by applying the *t*-distributed stochastic neighbor embedding (*t-SNE*) algorithm to the original feature vectors based on Continuous-TSSS. According to the coordinates of the same trinucleotides in Fig. 2, the uncertainty of feature vectors can be compared. For example, the coordinates of AAA in Fig. 2a is ($-0.97197$, 2.4612787), in Fig. 2b is ($-3.412198$, $-3.4761875$), and in Fig. 2c is (1.6702927, 2.9938223).

The feature vectors are significant to construct the ORI predictor, and the inner relationships among trinucleotides are of the worthiest to focus. It can be clearly seen that the feature vectors are divided into four regions in which trinucleotides have the same second nucleotide. In each region, it is obviously shown that the distance between two trinucleotides with different last nucleotide is very close, which is in line with the results of sequence segmentation. For example, TTA, TTC, TTG and TTT are respectively located in the left region of Fig. 2a, the lower region of Fig. 2b and the right region of Fig. 2c.

**Figure 3:**
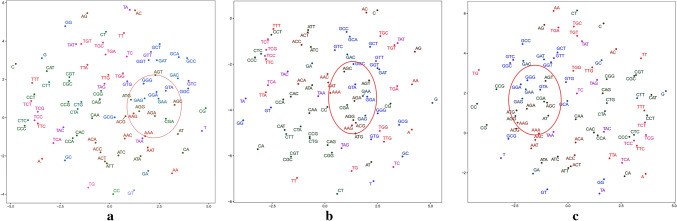
The 2-dimensional feature space generated by the *t-SNE* algorithm in the dataset $S_{1}$ Using Skip-TSSS. (**a**) The first training result. (**b**) The second training result. (**c**) The third training result.

On the basis of different sequence segmentation methods, the relative locations of words in feature spaces are reasonably different. Nonetheless, feature vectors trained by Word2vec based on Skip-TSSS can indicate the inner relationships of sequences. As shown in Fig. 3, it is intuitive that trinucleotides marked in each sub-figure are located close to each other but with quite different coordinates. For example, GAG, GGA, GAA are close to each other in Fig. 3a, but the distance between GGA and GAA are shorter than that between GGA and GAG. In the other sub figures, similar conclusions can be obtained.

The analysis above intuitively proves that the feature vectors constructed in this paper can effectively characterize the inner relationship among trinucleotides in ORIs. Subsequent experiments can further demonstrate that the method of constructing feature vectors plays an essential role in identifying ORIs from non-ORIs.

### Performance analysis of different models with Continuous-TSSS

To compare the performance of the proposed predictor under different modes, Table 1 lists identification results of each species based on Continuous-TSSS.

**Table 1:** The identification results of the proposed method based on different modes with Continuous-TSSS.

Dataset	Training mode	Acc	Sp	Sn	MCC	AUC
*S. cerevisiae* ($S_{1}$)	Default mode	0.920	0.923	0.917	0.840	0.921
	Embedding training mode	0.927	0.917	0.938	0.955	0.965
	Two channel mode	0.938	0.944	0.932	0.876	0.961
*S. pombe* ($S_{2}$)	Default mode	0.728	0.698	0.758	0.457	0.719
	Embedding training mode	0.719	0.727	0.711	0.439	0.721
	Two channel mode	0.754	0.721	0.787	0.510	0.770
*K. lactis* ($S_{3}$)	Default mode	0.885	0.912	0.858	0.771	0.888
	Embedding training mode	0.882	0.905	0.858	0.764	0.906
	Two channel mode	0.777	0.838	0.716	0.558	0.788
*P. pastoris* ($S_{4}$)	Default mode	0.956	0.958	0.954	0.911	0.967
	Embedding training mode	0.962	0.974	0.951	0.925	0.973
	Two channel mode	0.967	0.974	0.960	0.934	0.981

As can be seen from Table 1, if the training mode is set as the default mode, the *Acc*s achieved by the proposed predictor in the dataset $S_{1}$, $S_{2}$, $S_{3}$ and $S_{4}$ are 0.920, 0.728, 0.885 and 0.956, respectively. Except the *Acc*, the values of other measure indexes are satisfactory. Astonishing values in *AUC* are obtained, which are 0.921, 0.719, 0.888 and 0.967. The study also have achieved outstanding scores in *Sn* and *Sp*, which are 0.917, 0.758, 0.858, 0.954 and 0.923, 0.698, 0.9121, 0.958 respectively. Besides, the *MCC* was reached 0.840, 0.457, 0.771, 0.911, which reflects the high confidence of the proposed predictor derived by comprehensively considering the identification ability of positive and negative samples. These results indicate that the proposed predictor is completely feasible and reliable to identify ORIs. Critically, the identification results in the dataset $S_{2}$ do not meet the expectation.

What we expect is that the identification results with the other two modes should be better than those with the default mode. It is noticing that the overall identification results do not change much. If the training mode is set as ‘embedding training’ in the dataset $S_{3}$, each index changes from 0.728, 0.698, 0.758, 0.457, 0.719 to 0.719, 0.727, 0.711, 0.439, 0.721 respectively. In the dataset $S_{1}$, $S_{2}$ and $S_{4}$, there are slight improvements for each measure index. To summarize, the performance of the embedding training mode changes to some extent. It is possible that the feature vectors constructed in the dataset $S_{3}$ can reasonably characterize the ORIs. Therefore, the effects of feature vectors representation will be reduced when embedding layer is updated. Nonetheless, in the dataset $S_{1}$, $S_{2}$, $S_{4}$, the update of the embedding layer can make the effects of feature vector representation better, which provides a better performance of the proposed predictor in distinguishing ORIs from non-ORIs. If the training model is set as ‘Two Channel’, the values of measure indexes are greatly improved by comparing *Acc*, *MCC* and *AUC*, except in the dataset $S_{3}$. This is probably because the proposed model can obtain more appropriate inner features by updating embedding channel-2, meanwhile remaining the original features with unchanged embedding channel-1.

**Figure 4:**
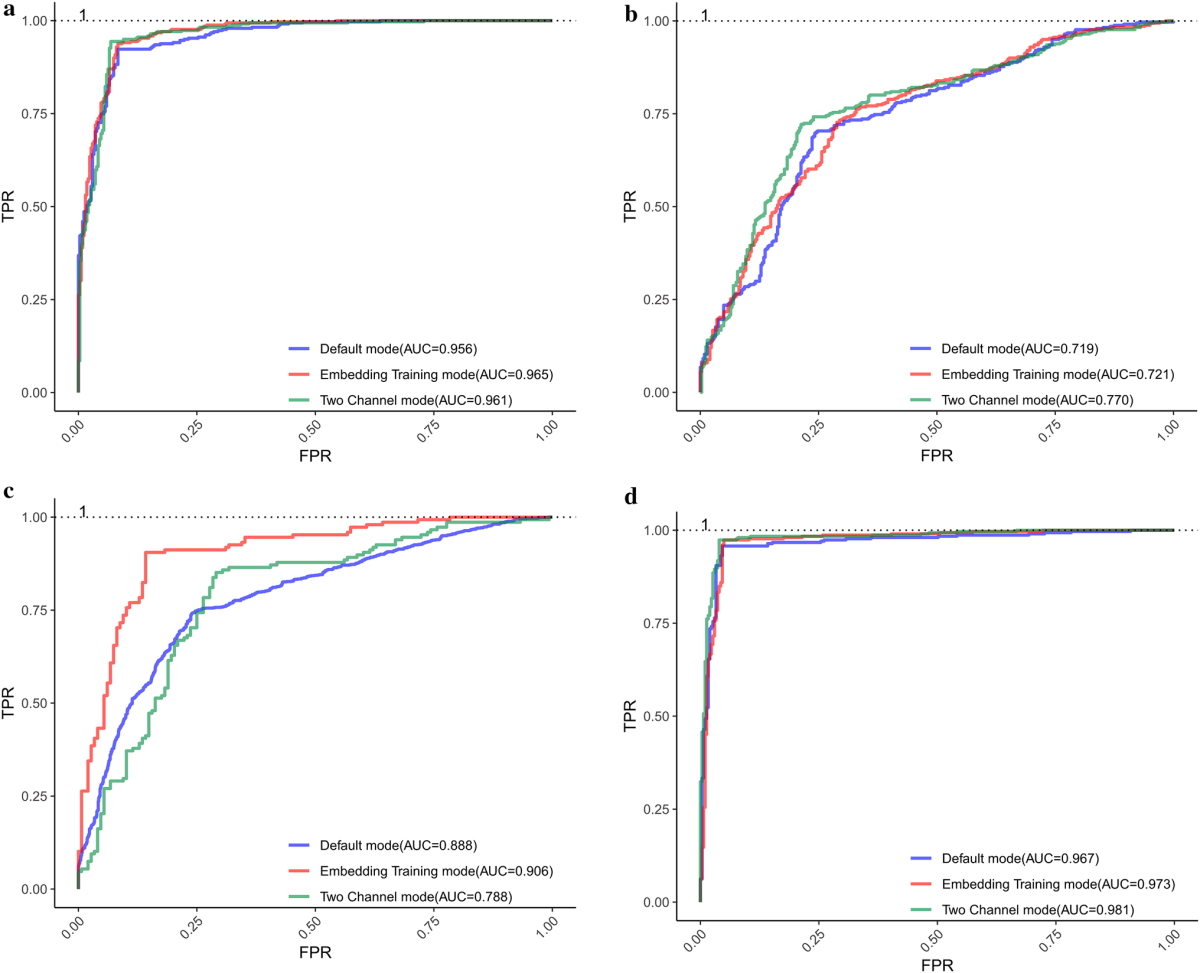
ROC curves obtained by using different training modes in each dataset based on Continuous-TSSS. (**a**) ROC curves of $S_{1}$. (**b**) ROC curves of $S_{2}$. (**c**) ROC curves of $S_{3}$. (**d**) ROC curves of $S_{4}$.

The *ROC* curves of different modes for each dataset are displayed in Fig. 4. It can be obviously seen that the proposed predictor performs pretty well and the *AUC* value of each *ROC* curves based on each training mode in the same dataset is different with each other, which can provide an intuitive comparison to find the best model to identify ORIs. The reasons for the excellent performance of the proposed predictor can be concluded as follows. Firstly, the deep relationships within trinucleotides and the biological significance of each trinucleotide are dug by Word2vec. Furthermore, the order information of trinucleotides is clearly reflected in the embedding layer, which enriches the features of ORIs. Finally, the deep network architecture is designed to conduct in-depth mining of sequence features, and an appropriate size of the convolution kernel is obtained by combining with the sequence segmentation method.

### Performance analysis of different models with Skip-TSSS

In order to further investigate the ability of the proposed predictor, Table 2 lists the details of ORI identification results based on different modes.

**Table 2:** The identification results of the proposed method based on different modes with Skip-TSSS.

Dataset	Training mode	Acc	Sp	Sn	MCC	AUC
*S. cerevisiae* ($S_{1}$)	Default mode	0.969	0.962	0.976	0.939	0.990
	Embedding training mode	0.975	0.966	0.983	0.940	0.975
	Two channel mode	0.972	0.962	0.981	0.944	0.972
*S. pombe* ($S_{2}$)	Default mode	0.731	0.741	0.720	0.461	0.754
	Embedding training mode	0.751	0.742	0.760	0.502	0.785
	Two channel mode	0.765	0.780	0.750	0.530	0.800
*K. lactis* ($S_{3}$)	Default mode	0.867	0.894	0.840	0.735	0.903
	Embedding training mode	0.857	0.872	0.842	0.714	0.891
	Two channel mode	0.811	0.824	0.797	0.622	0.852
*P. pastoris* ($S_{4}$)	Default mode	0.933	0.946	0.921	0.866	0.968
	Embedding training mode	0.956	0.964	0.948	0.912	0.972
	Two channel mode	0.949	0.964	0.933	0.898	0.972

As shown in Table 2, the proposed predictor performs well in each dataset no matter what the training mode is. In the dataset $S_{1}$, the identification results are improved from 0.938, 0.944, 0.932, 0.876, 0.961 (the best in Table 1) to 0.972, 0.962, 0.981, 0.944, 0.972 (the best in Table 2). For the dataset $S_{2}$, we gain a new best model based on the two channel mode, and achieve desired improvements by comprehensively considering indexes. Specifically, the *Acc*, *MCC* and *AUC* is improved from 0.754 to 0.765, 0.510 to 0.530, and 0.770 to 0.800 respectively. Meanwhile considering *Sp* and *Sn* together, the new model based on two channel mode performs a better stability. Nonetheless, in the dataset $S_{3}$ and $S_{4}$, the Skip-TSSS is not working as expected. Although the ORI identification results fluctuate up and down in other datasets, the overall performance of the proposed predictor is not out of our acceptable range. It can be concluded that the Skip-TSSS can significantly improve the accuracy of classifying ORIs from non-ORIs.

**Figure 5:**
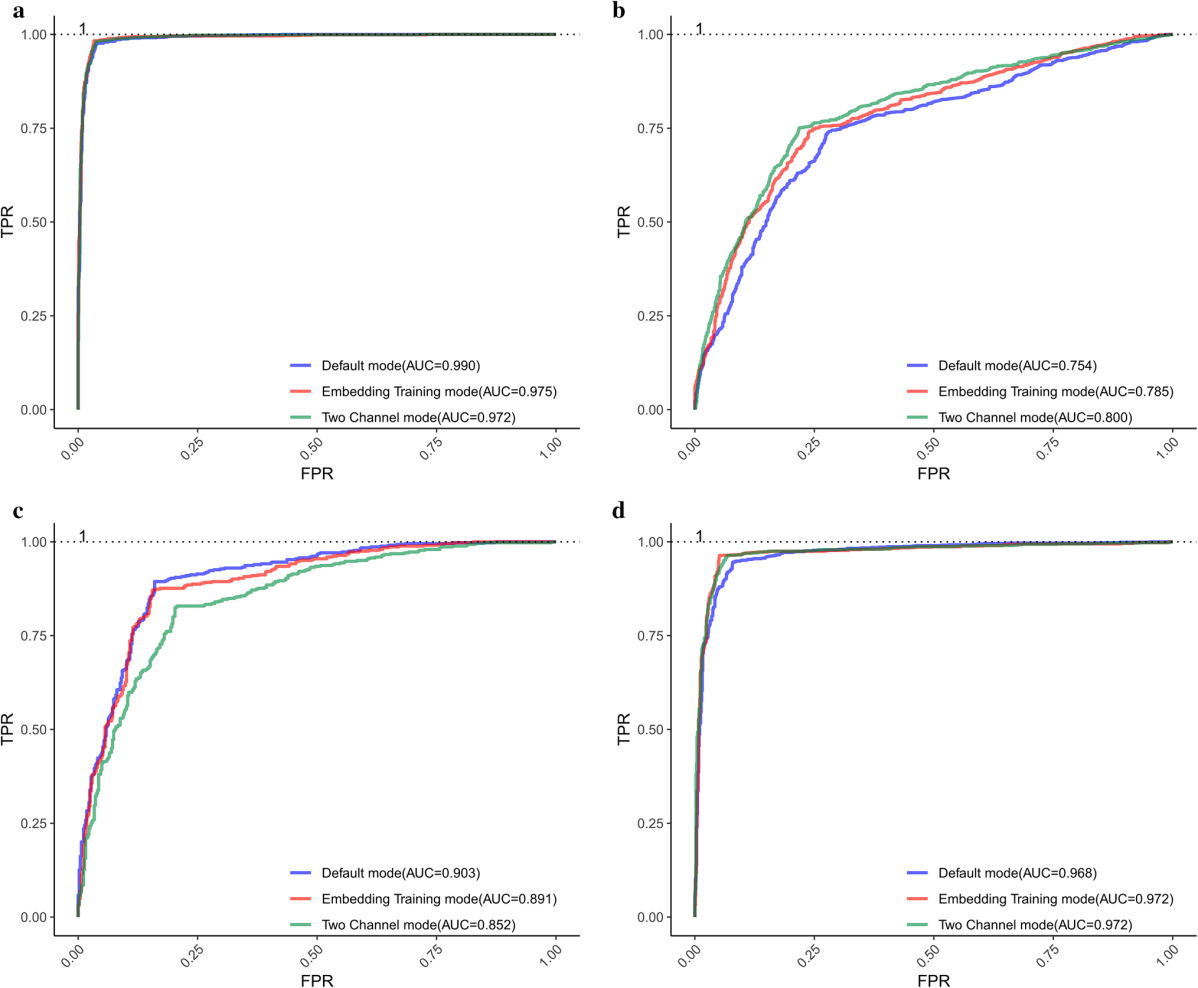
ROC curves obtained by using different training modes in each dataset based on Skip-TSSS. (**a**) ROC curves of $S_{1}$. (**b**) ROC curves of $S_{2}$. (**c**) ROC curves of $S_{3}$. (**d**) ROC curves of $S_{4}$.

In addition to the listed values of each measure index, the corresponding graph of *ROC* is obtained as shown in Fig. 5, where each parameter is the same as that in Fig. 4. In general, the proposed model used in each dataset based on three training modes performs well, which indicates that the performance of the model can be improved to some extent by selecting the best training mode.

### Prediction performance under the independent dataset test

In order to validate the generalization ability of the proposed method, 90% of the positive and negative samples are randomly selected as the training set and the remaining 10% as the independent test set. After the predictor is completely trained using the training set, the independent testing is performed using the independent test set. The independent dataset test is conducted 20 times and the corresponding performance measure indexes are averaged to avoid overfitting. Experimental results show that the prediction performance on the independent dataset as given in Table 3 is comparable to that on the training dataset as given in Tables 1 and 2 of the manuscript, indicating the robustness and the excellent generalization ability of the proposed method.

**Table 3:** The prediction performance of the proposed method under the independent dataset test.

Sequence segmentation	Dataset	Training mode	Acc	Sp	Sn	MCC
Continuous-TSSS	*S. cerevisiae* ($S_{1}$)	Default mode	0.9559	0.9697	0.9429	0.9122
		Embedding training mode	0.9412	0.9697	0.9143	0.8840
		Two channel mode	0.9559	1.0000	0.9143	0.9155
	*S. pombe* ($S_{2}$)	Default mode	0.8116	0.8788	0.7500	0.6315
		Embedding training mode	0.7826	0.8182	0.7500	0.5682
		Two channel mode	0.8116	0.9091	0.7222	0.6389
	*K. lactis* ($S_{3}$)	Default mode	0.9000	0.8462	0.9412	0.7964
		Embedding training mode	0.8667	0.8462	0.8824	0.7285
		Two channel mode	0.8333	0.8182	0.8421	0.6495
	*P. pastoris* ($S_{4}$)	Default mode	0.9500	0.9444	0.9583	0.8971
		Embedding training mode	0.9167	0.9167	0.9167	0.8281
		Two channel mode	0.9032	0.8947	0.9167	0.8009
Skip-TSSS	*S. cerevisiae* ($S_{1}$)	Default mode	0.9901	0.9794	1.0000	0.9804
		Embedding training mode	0.9901	0.9897	0.9906	0.9803
		Two channel mode	0.9951	0.9897	1.0000	0.9902
	*S. pombe* ($S_{2}$)	Default mode	0.7282	0.7353	0.7212	0.4564
		Embedding training mode	0.7379	0.6176	0.8558	0.4880
		Two channel mode	0.7573	0.7843	0.7308	0.5157
	*K. lactis* ($S_{3}$)	Default mode	0.9213	0.8667	0.9773	0.8482
		Embedding training mode	0.9213	0.8667	0.9773	0.8482
		Two channel mode	0.8539	0.9111	0.7955	0.7120
	*P. pastoris* ($S_{4}$)	Default mode	0.9454	0.9318	0.9579	0.8907
		Embedding training mode	0.9727	0.9659	0.9789	0.9453
		Two channel mode	0.9617	0.9432	0.9789	0.9238

### Comparison with the existing methods

In recent years, a series of machine learning-based methods have been proposed to identify ORIs for different species. To improve the effects of extracted features, Dao et al. employed two feature selection methods to develop an ORI predictor called ‘iORI-PseKNC2.0’^28^. Based on XGBoosting and Fasttext, Do et al. developed an ORI predicter for *Saccharomyces cerevisiae*^32^. By incorporating the ‘GC asymmetry bias’ into the ORI identification, Liu et al. developed a predictor called ‘iRO-3wPseKNC’ for four yeast species^35^. For further improving the prediction performance of iRO-3wPseKNC, they proposed a method called ‘iRO-PseKGCC’ to capture the ‘GC asymmetry bias’ of the gene sequences by considering both the GC skew and the sequence order effects of k-kuple GC composition^36^. To evaluate the performance of the proposed method more objectively, we chose the latest predictors mentioned above to make a better comparison with our method on the same benchmark datasets for corresponding four yeast species.

**Table 4:** Comparisons of the proposed method with the latest methods.

Species	Method	Acc	Sp	Sn	MCC	AUC
*S. cerevisiae* ($S_{1}$)	iRO-3wPseKNC^35^	0.730	0.752	0.707	0.459	0.808
	iRO-PseKGCC^36^	0.764	0.781	0.739	0.530	0.813
	iORI-PseKNC2.0^28^	0.782	0.802	0.763	0.565	0.831
	XGBoosting based method^32^	0.895	0.938	0.852	0.793	–
	The proposed method	0.975	0.966	0.983	0.940	0.975
*S. pombe* ($S_{2}$)	iRO-3wPseKNC^35^	0.965	0.949	0.979	0.929	0.986
	The proposed method	0.765	0.780	0.750	0.530	0.800
*K. lactis* ($S_{3}$)	iRO-3wPseKNC^35^	0.851	0.845	0.858	0.703	0.901
	The proposed method	0.885	0.912	0.858	0.771	0.888
*P. pastoris* ($S_{4}$)	iRO-3wPseKNC^35^	0.710	0.723	0.699	0.422	0.796
	iRO-PseKGCC^36^	0.742	0.739	0.745	0.484	0.800
	The proposed method	0.967	0.974	0.960	0.934	0.981

As listed in Table 4, the *Acc* achieved by several predictors in the dataset $S_{1}$ are less than 0.9, while our approach obtains an *Acc* of 0.975. Meanwhile, the *Sp* of 0.966 and *Sn* of 0.983 are also far better than those of the other methods. Moreover, a high score of *MCC*, 0.940, reflects a high confidence coefficient of prediction results. Similar to the dataset $S_{1}$, our method performs fairly well in the dataset $S_{4}$. Specifically, the *Acc*, *Sp*, *Sn* and *AUC* of the proposed predictor are more than 0.2 higher than those of the latest predictors. Especially, the *AUC* value can reach a level extremely close to 1. In the dataset $S_{3}$, though the *AUC* of the proposed predictor is 0.888, a little lower than that of iRO-3wPseKNC, our method shows a better performance in *Acc*, *Sp*, *Sn*, *MCC* with 0.885, 0.912, 0.858, 0.771, which can prove that our predictor can realize a stable identification of ORIs and non-ORIs with a high confidence coefficient. In the dataset $S_{2}$, the ORI identification results achieved by our method are not ideal, inferior to those achieved by iRO-3wPseKNC, which are exactly what needs to be addressed in the follow-up study.

In the recent publication^51^ , RFs trained on word2vec-derived encodings show unsatisfactory performance on bacterial ORIs. In this study, the word2vec combined with 2D-CNN achieves excellent identification results on eukaryotic ORIs. We will explain the contradictory results from a biological perspective.

**Figure 6:**
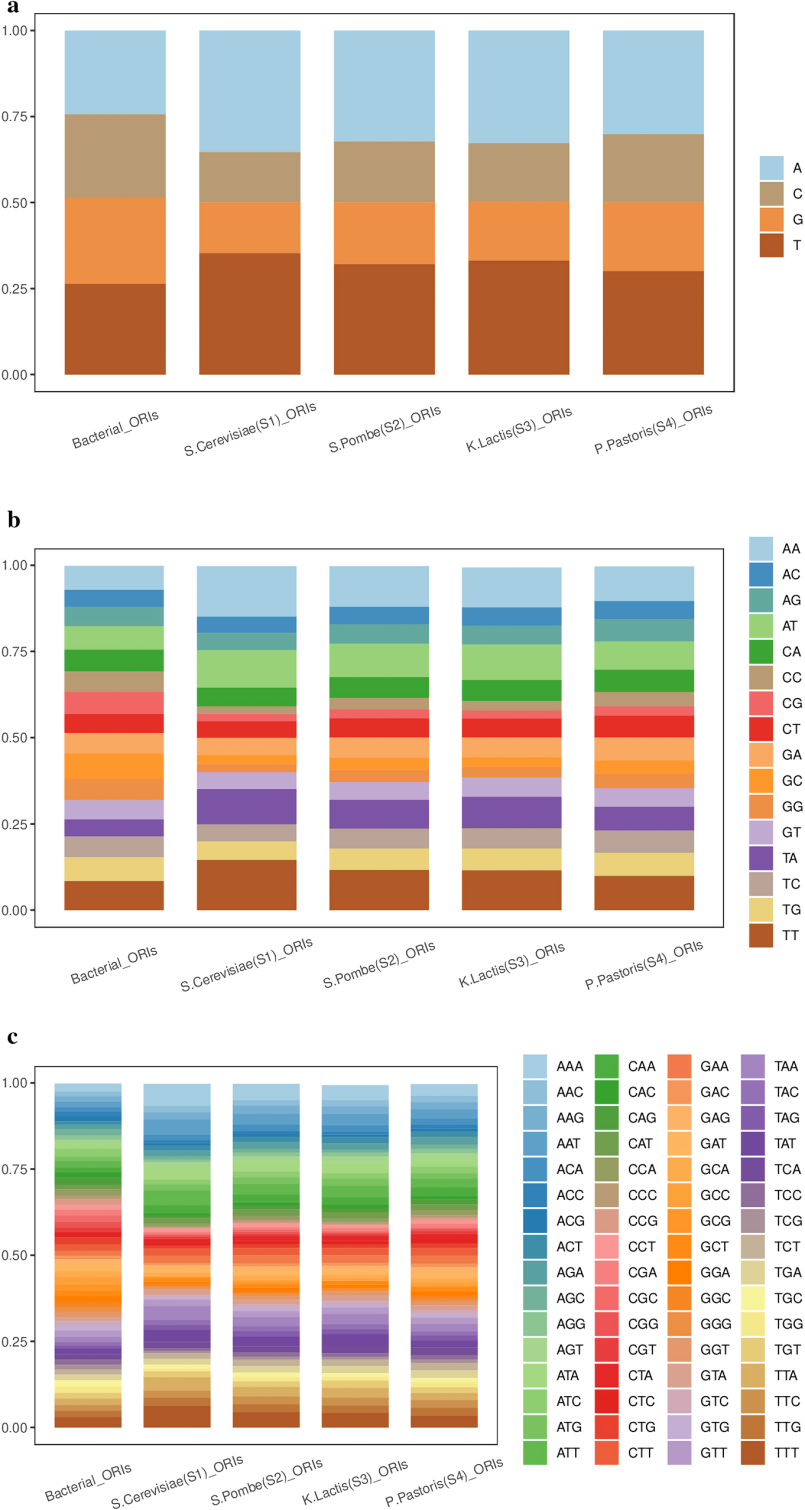
Nucleotide compositions in the flanking sequences of eukaryotic ORIs and bacterial ORIs. (**a**) Average mononucleotide frequencies in the flanking sequences of eukaryotic ORIs and bacterial ORIs. (**b**) Average dinucleotide frequencies in the flanking sequences of eukaryotic ORIs and bacterial ORIs. (**c**) Average trinucleotide frequencies in the flanking sequences of eukaryotic ORIs and bacterial ORIs.

Under different ecological and geographical conditions, the biological genes of different populations undergo genetic variations such as mutations, selections, and random drifts, leading to changes in the population gene frequency and genotype frequency. With the continuation of biological evolution, the accumulation of such changes will produce new species. Therefore, there might be a difference in evolutionary conservation between eukaryotic ORIs and bacterial ORIs. To analyze the general sequence-based evolutionary conservation of eukaryotic ORIs and bacterial ORIs, we calculate several statistical features in the flanking sequences of eukaryotic ORIs and bacterial ORIs. As shown in Fig. 6, there are big differences in terms of mononucleotide composition, dinucleotide composition and trinucleotide composition between the flanking sequences of eukaryotic ORIs and bacterial ORIs, which may be the reason for the different findings of the recent publication^51^ and this study. The flanking sequences of bacterial ORIs constructed by Sperlea et al.^51^ can be available in the supplementary file.

## Materials and methods

### Benchmark datasets

A reliable, stringent and comprehensive benchmark dataset is significant to the development of powerful methods for ORI identification. In this study, the genomic locations of replication origins and corresponding gene sequences for *Saccharomyces cerevisiae* (*S. cerevisiae*), *Schizosaccharomyces pombe* (*S. pombe*), *Kluyveromyces lactis* (*K. lactis*) and *Pichia pastoris* (*P. pastoris*) are derived from DeOri6.0^52^ and GenBank^53^, respectively. DeORI6.0 is a database of Eukaryotic ORIs, which contains all the eukaryotic ones identified by genome-wide analyses. GeneBank is a gene sequence database, which brings together and annotates all publicly available nucleic acid and protein sequences. Each record represents a single, continuous, annotated piece of DNA or RNA. The gene sequences with ORIs are taken as positive samples. The negative samples in each dataset are randomly extracted from the gene sequences of corresponding yeast species without ORIs, and have the same length distribution as the positive samples. The DNA fragments with the length less than 50 dp are deleted. To remove redundant samples and reduce homologous bias, the CD-HIT software^54^ is used to delete sequences with sequence similarity greater than 80% (the setting of 80% can approach the balance to some extent between maintaining the statistical significance of the sequences and avoiding overfitting) to any other sequences in each dataset. To avoid the data imbalance, the difference between the number of positive samples and negative samples in each dataset are not supposed to be too large. Finally, four benchmark datasets are constructed as

1 $$\begin{aligned} S = S^+\bigcup S^- = \left\{ \begin{aligned} S^{+}_{1} \bigcup S^{-}_{1}&(S_1, S. cerevisiae)\\ S^{+}_{2} \bigcup S^{-}_{2}&(S_2, S. pombe)\\ S^{+}_{3} \bigcup S^{-}_{3}&(S_3, K. lactis)\\ S^{+}_{4} \bigcup S^{-}_{4}&(S_4, P. pastoris) \end{aligned} \right. \end{aligned}$$

where $S_{1}$ contains 340 ORI sequences and 342 non-ORI sequences. $S_{2}$ contains 342 ORI sequences and 338 non-ORI sequences. $S_{3}$ contains 147 ORI sequences and 147 non-ORI sequences. $S_{4}$ contains 305 ORI sequences and 302 non-ORI sequences. The $\bigcup$ represents the union symbol in the set theory.

### Sequence segmentation

#### Continuous-three slices sequence segmentation

The process of the gene information transmission and gene expression is mainly divided into the sub-processes of transcription, translation, and the synthesis of the functional protein. These sub-processes all take ‘three’ as the basic size of an operation unit, such as codon, anticodon and the amino acids carried by tRNA. Thus, in order to maintain the integrity of the biological basis, the higher grams are not considered. Purely in terms of the sequence segmentation method, as for $n > 3$, the dimensions of word vectors increase dramatically, and may introduce some redundant information. In view of this, a sequence segmentation method called ‘Continuous-Three Slices Sequence Segmentation’ (Continuous-TSSS) is utilized in this paper.

A gene sequence is denoted as:

2 $$\begin{aligned} D=R_{1}R_{2}R_{3}\cdots R_{i-1}R_{i}R_{i+1} \cdots R_{L-2}R_{L-1}R_{L}, \end{aligned}$$

where *L* represents the length of the gene sequences; $R_{i}$ represents the *i*th nucleotide of the gene sequence. Based on the window with the size fixed to 3, the trinuleotides inside the parentheses of Eq. (3) can be obtained by moving with the stride size set as 1 from the beginning of the gene sequence.

3 $$\begin{aligned} (R_{1}R_{2}R_{3}), (R_{2}R_{3}R_{4})\cdots (R_{L-2}R_{L-1}R_{L}) \end{aligned}$$

#### Skip-three slices sequence segmentation

**Figure 7:**
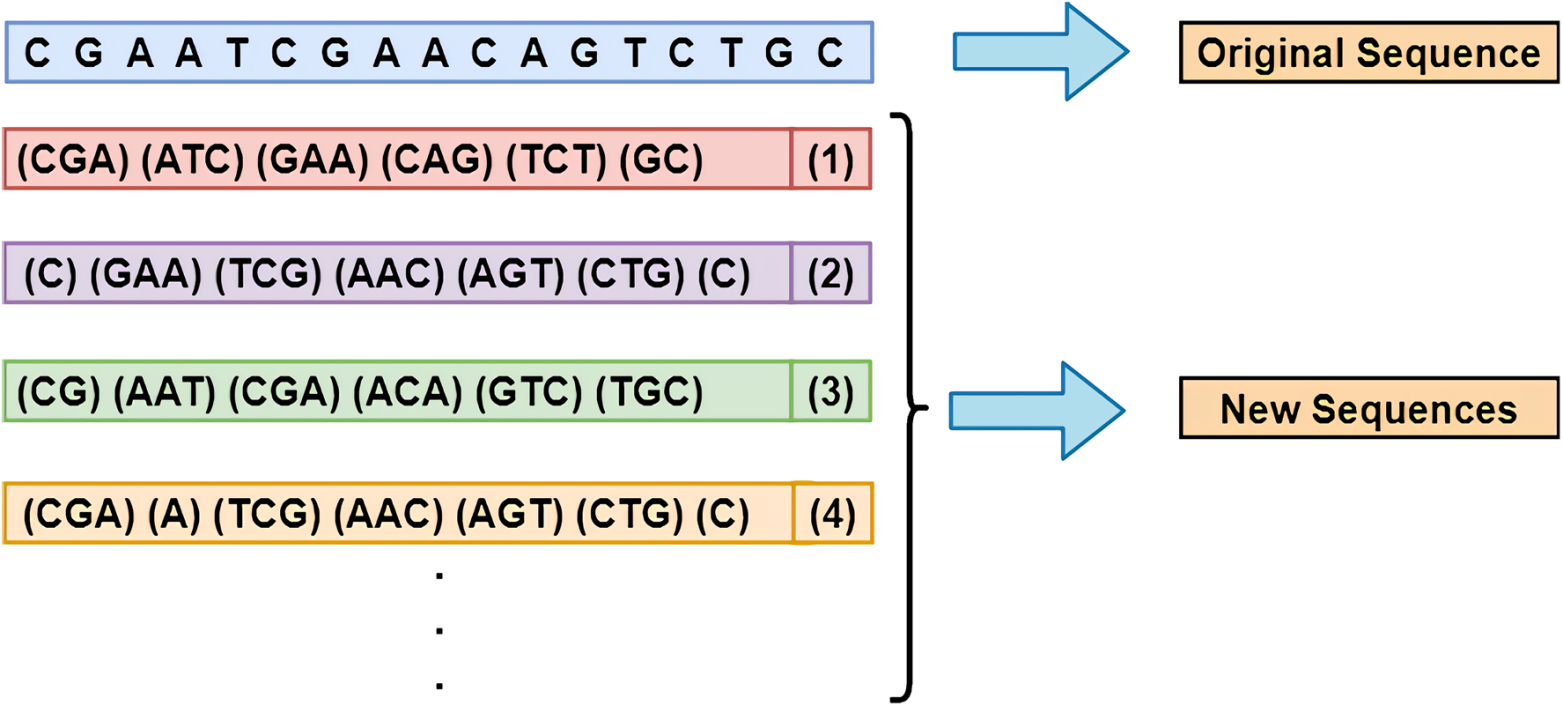
The process of Skip-three slices sequence segmentation (Skip-TSSS).

As mentioned in “Skip-three slices sequence segmentation” section, the Continuous-TSSS, a window with size of 3 moves on a sequence with step size of 1, can not entirely extract composition features of several nucleotides in head and tail of sequences compared to other nucleotides. In view of this, each nucleotide is needed to be reused to fully extract the characteristic of each nucleotide in original sequences. Another sequence segmentation method called ‘Skip-Three Slices Sequence Segmentation’ (Skip-TSSS) is developed in this study to split a gene sequence into three sequences, which changes the subsequent construction of feature vectors but can remain the original gene sequence order at the same time. Using a window of size 3 to move from the beginning of a sequence with a step size of 3, a new sequence is obtained without deleting any nucleotide from the original sequence. If the length of the original sequence is divisible by 3, the new sequence entirely consists of trinucleotides. If the length of the original sequence divided by 3 with a remainder of 1, the last component of the new sequence is a mononucleotides. In addition to the above two cases, the last unit of the new sequence is a dinucleotide. Similarly, if the window moves on the original sequence starting at different positions, more sequences can be obtained as shown in Fig. 7.

If the window keeps moving on the original sequence, the new sequences generated by the Skip-TSSS will have a high similarity with the first three sequences. As an example in Fig. 7, the differences between the second and fourth new sequences are only reflected on the first and second component units, which can prove that the similarity between these two sequences is extremely high, because of the length of a sequence may reach more than 3000 dp even 10,000 dp. To avoid highly repetitive sequences and preserve the biological significance of the new sequences, the original sequences are segmented into the first three new sequences without any cuts. It is worth noticing that three sequences generated from one original sequence are completely different from each other, but they can represent the property of the original sequence. In brief, the new sequences generated by the Skip-TSSS is made up of 4 types of mononucleotides, $4\times 4=16$ types of dionucleotides, and $4^{3}=64$ types of trinucleotides.

### Feature vector construction

As one of the classical word vector training models in the field of NLP, the skip-gram model of Word2vec^55^ based on the negative sampling^56^ is adopted in this study to construct the feature vectors of trinucleotides. Specifically, given a trinucleotide in any position of the gene sequence to be the central trinucleotide, the corresponding feature vector can be obtained by maximizing the probability of predicting its surrounding trinucleotides.

Word2vec was created with the goal of extracting strong correlation features that exist within the local interval of a text. The main idea is that the adjacent words are strongly related to each other and it is possible to infer from the context what the current vacant word is. Therefore, Word2vec can effectively extract advanced features in a small interval. Moreover, word2vec is the cornerstone of word vector training, which focuses only on the words in the current window, regardless of grammar, language structure, and multiple meanings of words. Objectively, biological sequences do not have, or humans have not yet found words, sentences, and grammars that are similar to those found in natural languages. Word2vec can avoid too much involvement with the properties of natural languages, thereby contributing to the interpretability of word vectors.

Firstly, the length of each trinucleotide is defined as:

4 $$\begin{aligned} len(w)= \frac{[counter(w)]^{\frac{3}{4}}}{\sum \limits _{i\in D}[counter(i)]^{\frac{3}{4}}}, \end{aligned}$$

where $counter(\cdot )$ represents the frequency of a trinucleotide; *D* represents a set of all the trinucleotides.

According to Eq. (4), the sum of the distribution length of trinucleotides is 1, thereby a non-uniform interval [0, 1] represents the original distribution of trinucleotides. After dividing [0, 1] into *M* parts equidistantly, a negative sample is obtained by randomly selecting $m_{n}$ and mapping it to the original non-uniform distribution as the following formula:

5 $$\begin{aligned} Table(n) = w_{k},\ m_{n} \in L_{k}, n = 1,2,\ldots , M-1, \end{aligned}$$

where $L_{k}$ represents the length of word $w_{k}$; $m_{n}$ represents the $n^{th}$ part in uniform distribution; *Table*(*n*) denotes that $m_{n}$ is mapped to the original non-uniform distribution to represent $w_{k}$.

A negative sampling sample can be derived by mapping the result of random sampling in the uniform distribution interval to the original non-uniform interval. The process of the negative sampling is terminated until the size of negative sampling set is in line with our requirements.

Based on the principles of the employed model, the target function is defined as:

6 $$\begin{aligned} G=max \left( \prod _{u\in Context(w)} \prod _{z \in \{u\}\cup NEG(u)}p(z|w) \right) , \end{aligned}$$

where *w* represents the central trinucleotide. *Context*(*w*) is denoted as the set of context of *w*. *NEG*(*u*) represents the set formed by the negative sampling of *u*. *z* represents the trinucleotide in the union of *NEG*(*u*) and *u*. *p*(*z*|*w*) is defined as the observation probability of each trinucleotide in the *Context*(*w*).

Equation 6 indicates that the calculations of probability values require the negative sampling set of each surrounding trinucleotide. To reduce the algorithmic complexity, all the negative samplings are operated on the central trinucleotides. Thus, the target function is modified as:

7 $$\begin{aligned} G=max \left( \prod _{\tilde{w}\in Context(w)} \prod _{u \in \{w\}\cup NEG^{\tilde{w}}(w)}p(z|w) \right) , \end{aligned}$$

where $\tilde{w}$ represents each trinucleotide in the set of surrounding trinucleotides. $NEG^{\tilde{w}}(w)$ represents the set formed by the negative sampling of the central trinucleotide *w* while using $\tilde{w}$ to predict *w*. *u* represents the trinucleotide in the union of $NEG^{\tilde{w}}(w)$ and *w* $p(z|\tilde{w})$, the probability of observing $\tilde{w}$, is defined as:

8 $$\begin{aligned} p(z|\tilde{w})=\left\{ \begin{array}{lcl} \sigma (v(\tilde{w})^{T}\theta ^{u}), &{} \quad L^{w}(u)=1 \\ 1 - \sigma (v(\tilde{w})^{T}\theta ^{u}), &{} \quad L^{w}(u)=0 \end{array} \right. \end{aligned}$$

where $v(\tilde{w})$ is defined as the vector of trinucleotide $\tilde{w}$; $\theta ^{u}$ represents the support vectors to calculate the score of binary classification with the same size of $v(\tilde{w})$; $\sigma (\cdot )$ represents the sigmoid fucntion to calculate probability limited in range of $[0,\ 1]$. Besides, $L^{w}(u)$ is the label of *u* as given in following equation. That is to say, the label of center trinucleotide *w* is 1, and the label of any non-central trinucleotide is 0.

9 $$\begin{aligned} L^{w}(u)=\left\{ \begin{array}{lcl} 1, &{} \quad u=w \\ 0, &{} \quad u\ne w \end{array} \right. \end{aligned}$$

In order to describe the training processes of word vectors in detail, a DNA sequence after word segmentation (ACG, CGT, GTC, **TCG**, CGT, GTA) is taken as an example. Assuming that the central trinucleotide is TCG and the range of context is set to 2, trinucleotides in the context is used to predict the probability of the central trinucleotide based on Eq. (7). Meanwhile, the value of *M* in the negative sampling process is set as 1000. While the prediction probability of TCG is calculated using CGT in context, the negative samplings are operated on the central trinucleotides TCG to obtain $NEG^{CGT}(TCG)$, and calculate the prediction probability according to Eq. (8). Similarly, when using other contexts to predict TCG, negative sampling of TCG is carried out respectively, and the probability is calculated after obtaining the negative sampling set. The error backpropagation, parameter update and word vector update are completed by maximizing prediction probability, and then the central word is moved to the next position. Generally, if the dimension is set be a small value, the information will be compressed or even covered; if the dimension is set to be a large value, much redundant information would be contained and the word vector is too sparse to depict the relationship between words. Due to the large range of the dimension, it is impractical to search optimal values of the dimension by the method of exhaustion. Thus, the proposed method is firstly constructed with the dimension chosen from a wide range, and then the search range around the best dimension is narrowed gradually. After many iterations, 300 that yields the best prediction performance is selected as the optimal dimension. Finally, on the basis of Continuous-TSSS, a pre-training feature matrix for ORI sequences is constructed with the dimension of $64\times 300$ after several training iterations based on the positive samples while the dimension of the feature matrix is $84\times 300$ on the basis of Skip-TSSS.

### Architecture of convolutional neural network

**Figure 8:**
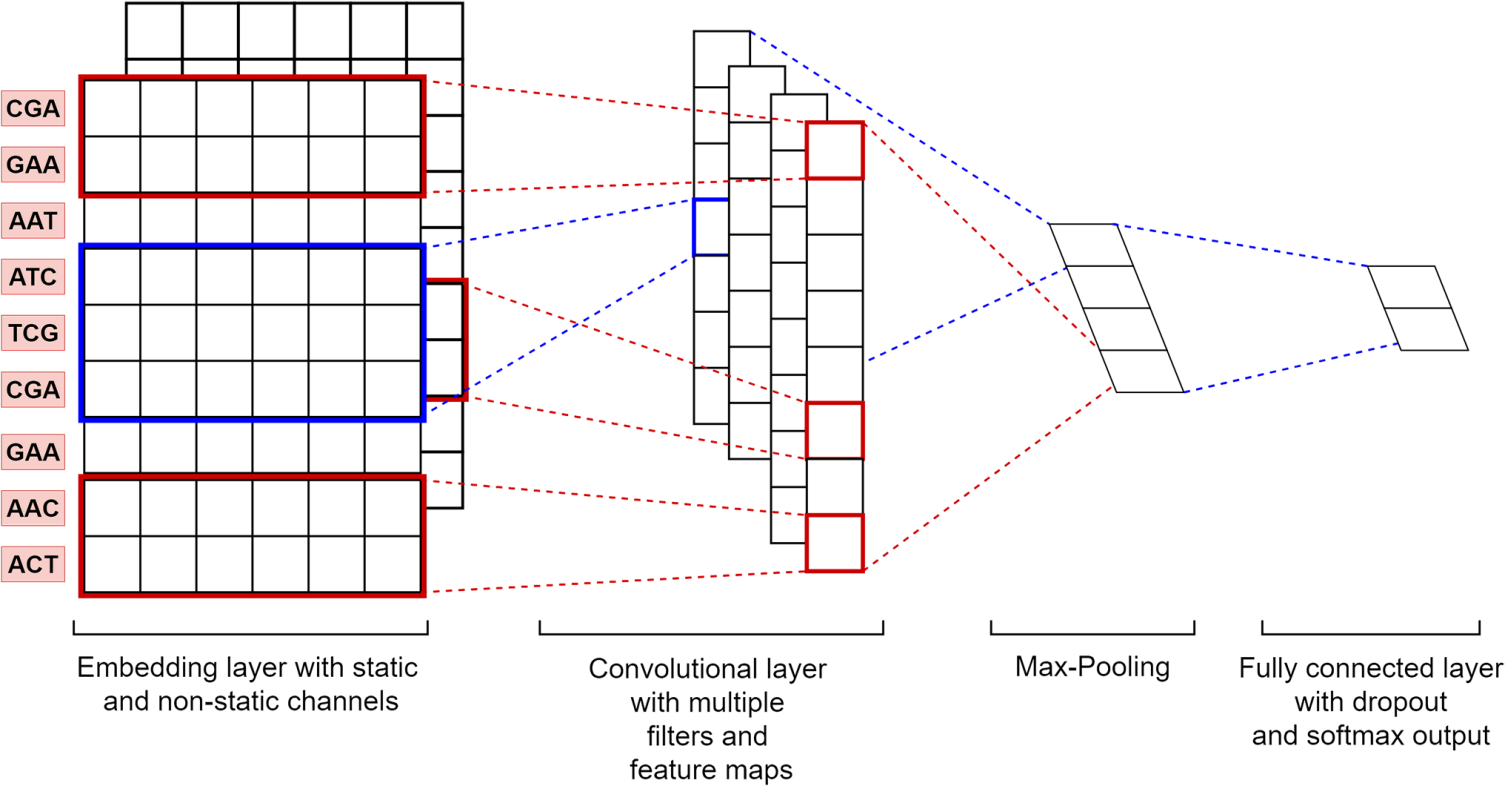
The architecture of the proposed CNN.

In the era of artificial intelligence, the abilities of the deep learning algorithm have been widely recognized. The CNN, the most representative deep neural network architecture, is widely used in the field of image processing and generally includes input layer, convolutional layer, pooling layer and output layer. Taking the pre-training feature matrix as the input, the architecture of CNN based on the Continuous-TSSS as shown in Fig. 8 will be explained in the following subsections.

#### Input layer

The input layer is constructed by vertically arranging the trinucleotides of the input sequence, which aims to treat each trinucleotide in the sequence as the object to be processed rather than the whole sequence.

#### Embedding layer

Given a sequence *x* of length *L*, it can be segmented into a new sequence *y* of length $L-2$. Assuming that the new sequence is composed of *N* kinds of trinucleotides, the dimension of the one-hot matrix *O* obtained by the one-hot encoding^57^ will be $N\times N$, and the dimension of the pre-trained trinucleotide feature matrix generated by Word2vec will be $N\times D$.

**Figure 9:**
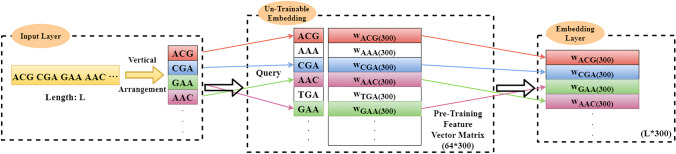
The un-trainable embedding layer construction.

As shown in Fig. 9, on the basis of the word order of the input sequence, pre-training feature vectors will be added to the corresponding lines of the embedding layer by matching each word in the input sequence with each row index in the embedding matrix.

**Figure 10:**
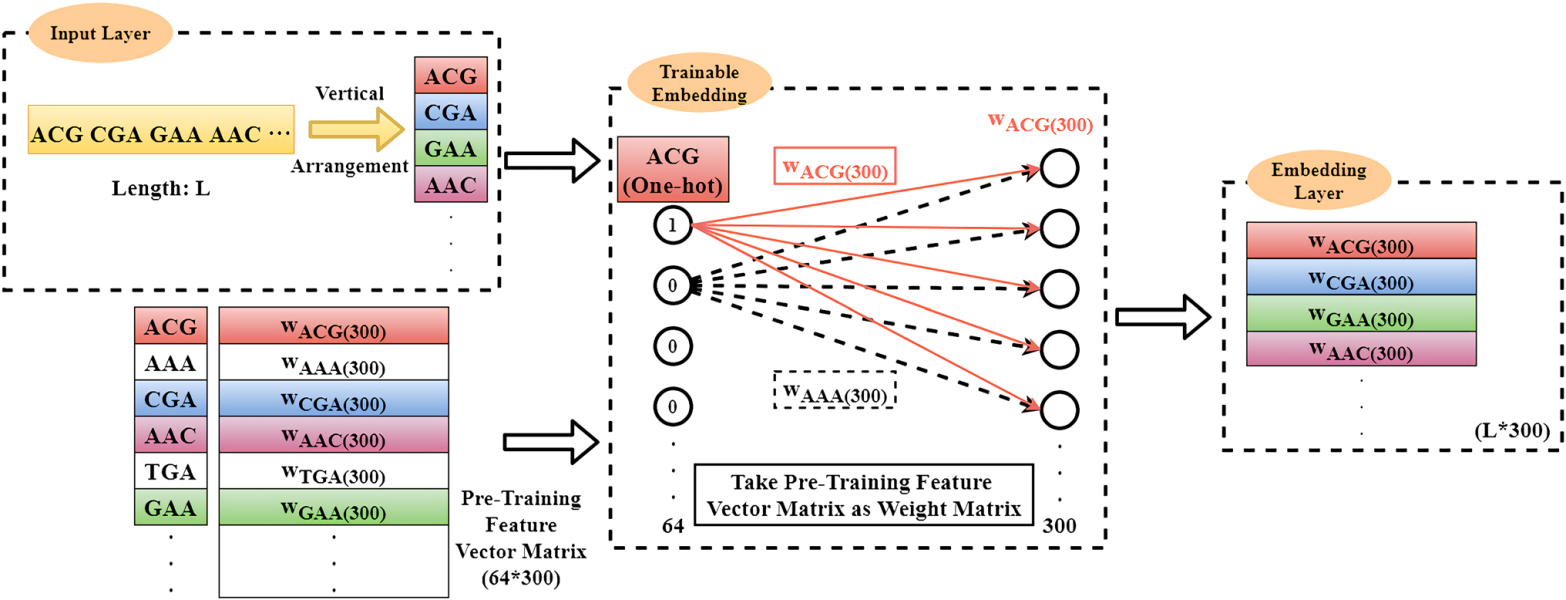
The trainable embedding layer construction.

If the input is the One-hot code of each trinucleotide and the weight matrix is the pre-trained trinucleotide feature matrix W obtained by Word2vec, the embedding layer can be regarded as the outputs of the fully connected layer, i.e. the trinucleotide vector of the corresponding position of the ith trinucleotide. As shown in Fig. 10, the training of the embedding layer is realized by the Back Propagation (BP) algorithm^58^.

As only one element of the one-hot vector is 1 and the rest are 0, just one neuron is activated at each input. Thus, there are just one group of weights activated to update corresponding row of embedding matrix *z*. The obtained trinucleotide vector has no differences from the trinucleotide vector in *W*, but the obtained embedding matrix is vertically arranged.

**Figure 11:**
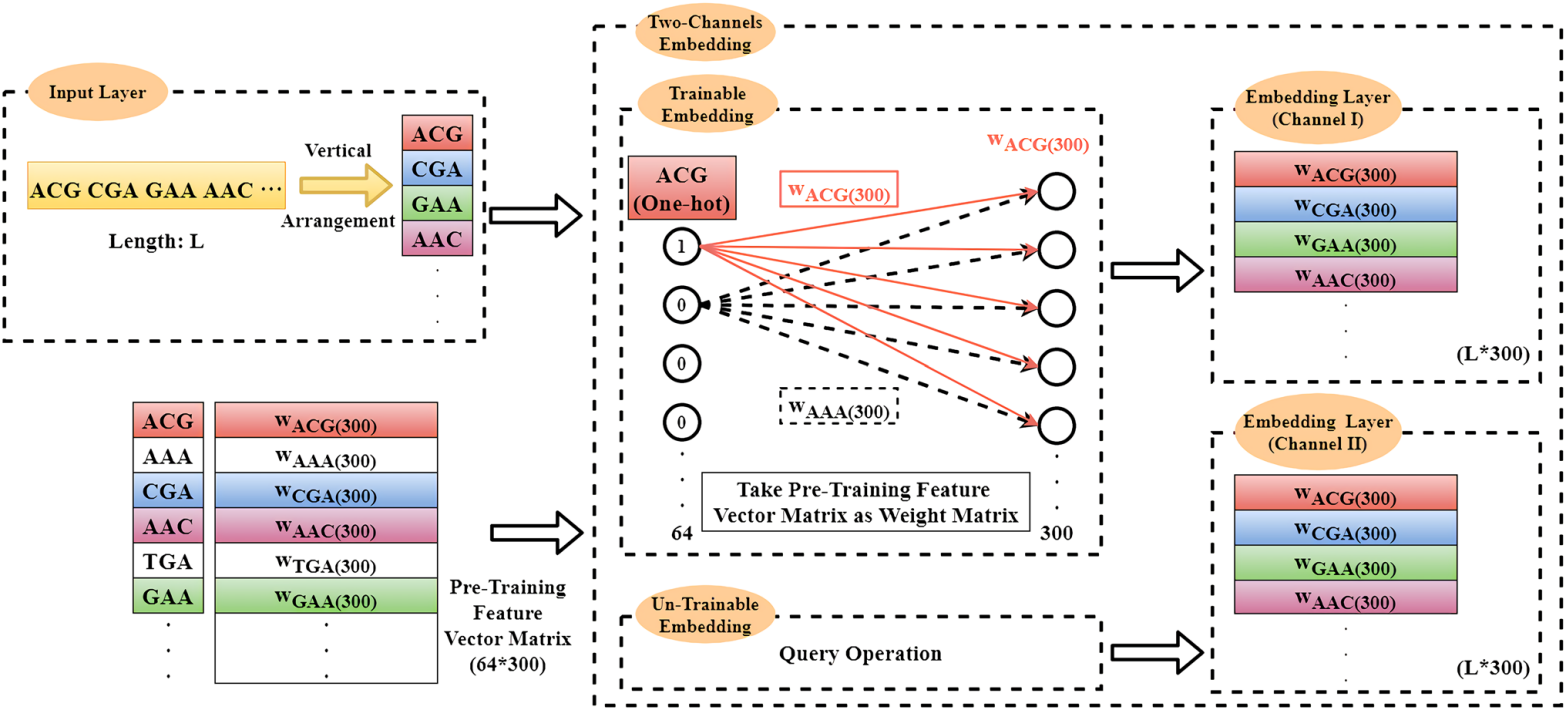
The two-channel embedding layer construction.

According to the aforementioned the embedding layer construction process, there are three modes utilized to train the network as described below:

- **The Default mode:**
  The embedding layer is set as ‘un-trainable’. In this case, the construction of the embedding matrix becomes a query operation or a matrix operation, i.e.
  10 $$\begin{aligned} z[R_{1},R_{2},\ldots ,R_{L-2}]\{D\}= W[y[R_{1},R_{2},\ldots ,R_{L-2}]]\{D\} \end{aligned}$$
  where *y* represents a DNA sequence with a length of *L*; $R_{i}$ represents the index of the $i^{th}$ trinucleotide; *D* is defined as the dimension of feature vectors; *z* represents the embedding matrix (or embedding layer); *W* represents the pre-trained trinucleotide vectors matrix; $z[R_i]\{D\}=W[y[R_i]]\{D\}$. For example, if $R_i=2$, the trinucleotide vector whose index is 2 in *W* is queried and added to the second row of *z*. Finally, an embedding matrix with a dimension of $(L-2)\times D$ can be formed.

- **The Embedding Training mode:**
  The embedding layer is set as ‘trainable’. In this case, according to the BP algorithm, the embedding matrix is updated with the weight matrix *W* in the fully connected layer.

- **The Two Channel mode:**
  The ‘Two Channel’ mode is similar to the RGB channels in the area of Image Processing. In this case, the number of channels is set to be 2. As shown in Fig. 11, one embedding layer is set as ‘trainable’ while the other one set as ‘un-trainable’ .

#### Convolutional layer

Although the output of the embedding layer is a matrix, horizontal convolution on the matrix is meaningless, due to the fact that each row of the matrix is the feature vector of the corresponding trinucleotide. For vertical convolution operation to extract the features among several rows, the width of the convolution kernel is set as the width of the embedding layer, and the height of the convolution kernel is set arbitrarily. By using convolution kernels with different sizes to carry out vertical convolution, more perceptive fields can be obtained to extract features in a wider range. At the same time, the convolution kernels can be initialized to different values, thereby obtaining more feature information in the same region.

**Figure 12:**
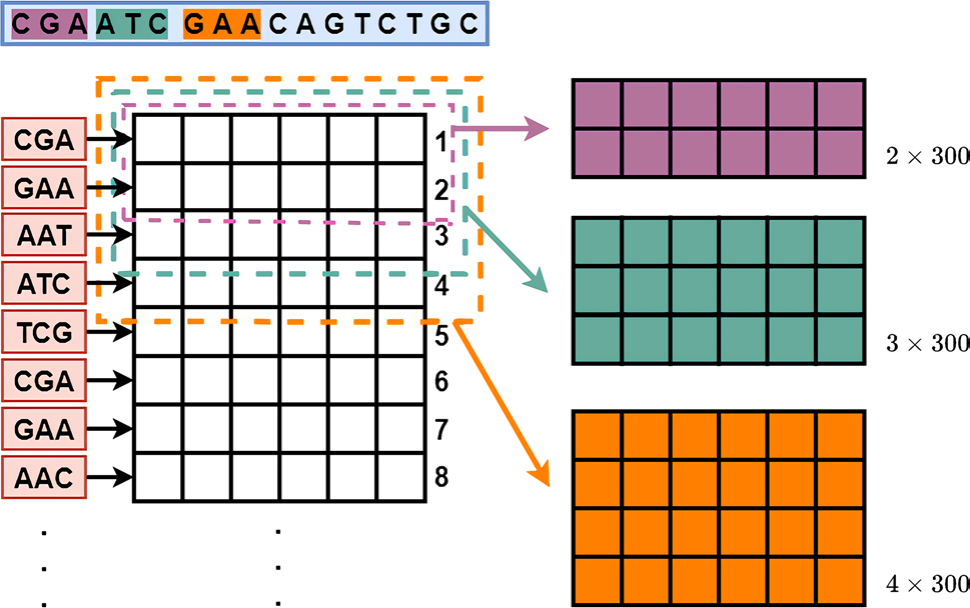
The sizes of the convolutional kernel employed in the CNN.

According to the ‘Continuous-TSSS’, the sequence after the word segmentation is composed of $L-2$ words. If the size of the convolution kernel is set to be 2, the feature information between the two words is extracted to represent the relationship between the current trinucleotide and the adjacent nucleotide in the original sequence. Similarly, if the size of the convolution kernel is set to be 3, the feature information between the three words is obtained to characterise the overall and partial relationship between the current trinucleotide and the adjacent dinucleotide in the original sequence. Analogously, if the size of the convolution kernel is set to be 4, the feature information of four consecutive words is fused, which is equivalent to the feature information between two adjacent trinucleotides in the original sequence. The sizes of the convolutional kernel to construct the CNN are illustrated in Fig. 12. To obtain more features of the same region, the number of the convolution kernel with each size is set as 128. Finally, $128 \times 3$ feature maps are obtained as the input of the pooling layer.

#### Pooling layer

In the Pooling layer, the downsampling is performed to reduce the dimensions of feature maps, thereby reducing the risk of overfitting and accelerating model training. In this paper, Max-Pooling is respectively employed to extract the maximum value from $128 \times 3$ feature maps, and then concatenate the Max-Pooling results to form a single feature vector with a dimension of $128 \times 3=384$.

#### Fully connected layer

To integrate feature information output by the Pooling layer, a fully connected layer is constructed to perform classification tasks with a size of $384$. In addition, the Softmax function is employed to calculate scores for a DNA sequence identified as ORI or non-ORI, and the final identification result is given with a higher score.

**Table 5:** The hyper-parameters of the proposed CNN based on the default mode.

Hyper-parameter	Value
Input length	$L_{max}$
Batch size	64
Embedding layer	$L_{max} \times 300$, *Trainable* = False,
	*Num*_*Channels* = 1
Convolution blocks	$[2,\ 3,\ 4]$, $128 \times 3$, ReLu
Pooling blocks	Max-pooling
Fully connected layer units	$128 \times 3$
Regularization	L2
Learning rate	0.001 with decay rate 0.9
Optimizer	Adam

Based on the Default Mode, the setting of hyper-parameters is listed in Table 5 where $L_{max}$ represents the longest length of the sequences; *Trainable* which can be set as ‘True’ of ‘False’ represents the parameter to determine whether the embedding layer can be trained; The *Num*_*Channels* is the parameter to represent the number of channels in the embedding layer. Embedding layer can realize the conversions from trinucleotides to corresponding pre-training feature vectors. In convolution blocks with an activation function of *ReLu*, convolutional kernels are set as $[2,\ 3,\ 4]$ respectively, and the number of kernels for each size is set to be 128. *L2* regularization is adopted to avoid over-fitting, which can ensure the availability of the proposed architecture. *Adam* is chosen as the optimizer to compute different and adaptive learning rates for each parameter using a batch size of 64 for an initial learning rate of 0.001 with a decay rate of 0.9.

### Performance evaluation

To evaluate the quality of the proposed predictor in a comprehensive and efficient way, it is necessary to set up a complete metrics system. In the area of statistical prediction, five measure indexes are widely used to evaluate predictors: the overall accuracy (*Acc*), the sensitivity (*Sn*), the specificity (*Sp*), the Matthew’s correlation coefficient (*MCC*), and the *AUC* (the Area Under the Receiver Operating Characteristic (*ROC*)^59^). Based on the symbols introduced by Chou^60^, the first four indexes can be defined as following formula:

11 $$\begin{aligned} Sn= & {} \dfrac{TP}{TP+FN}, \quad 0\le Sn\le 1 \end{aligned}$$

12 $$\begin{aligned} Sp= & {} \dfrac{TN}{TN+FP}, \quad 0\le Sp\le 1 \end{aligned}$$

13 $$\begin{aligned} Acc= & {} \dfrac{TP+TN}{TP+TN+FP+FN}, \quad 0\le Acc\le 1 \end{aligned}$$

14 $$\begin{aligned} MCC= & {} \frac{1-\left( {\frac{{FN}}{{TP+FN}}+\frac{{FP}}{{TN+FP}}}\right) }{\sqrt{\left( 1+\frac{{FP-FN}}{P}\right) \left( 1+\frac{{FN-FP}}{N}\right) }}, \quad -1\le MCC\le 1 \end{aligned}$$

where *TP* represents the number of ORIs that are correctly predicted. *TN* represents the number of non-ORIs that are correctly predicted. *FN* denotes the number of ORIs that are incorrectly predicted to be the non-ORIs. *FP* denotes the number of non-ORIs that are falsely predicted to be ORIs. *P* is defined as the total number of ORIs. *N* is defined as the total number of the non-ORI.

Note that, of the four metrics in Eqs. (11)–(14), the most significant are the *Acc* and *MCC*: the former reflects the overall accuracy of a predictor, while the latter represents the stability of a predictor in practical applications. Both *Sn* and *Sp* of the predictor A are higher than those of the predictor B, we can declare A is better than B. To make a more meaningful comparison, *MCC* is utilized to realize simple and convenient comparison.

In order to demonstrate the performance of a predictor intuitively, the *ROC* with $1 - Sp$ as the horizontal axis and the *Sn* as the vertical axis is used to evaluate the performance across the entire range of its decision values in this paper. In general, the *ROC* of a good predictor is above the line $y=x$. As the *x*-coordinate increasing, the *y*-coordinate approaches 1, which means better performance. *AUC* is the area under the *ROC* curve, $0<AUC<1$. The closer the *AUC* is to 1, the better the performance of the predictor is.

### K-fold cross validation

With the metrics system to evaluate the quality of a predictor, it is necessary to utilize a reasonable validation method to score five measure indexes. The 10-fold cross validation is employed in this study that divides the dataset into 10 parts, among which just one part is used as the test set in turn, and nine parts are taken as the training set for experiments. Each fold of experiment can obtain the *TP*, *TN*, *FP*, *FN*, and the probability of predicting each sample in the test set. After the experiments, ten sets of such data can be obtained, and the corresponding evaluation indexes can be calculated. Meanwhile, the *ROC* curve can be drawn based on the probability of each sample, which can better reduce the experiment errors and randomness.

## Supplementary Information

Supplementary Information.
